# Normal Electrical Activity of the Brain in Obsessive-Compulsive Patients After Anodal Stimulation of the Left Dorsolateral Prefrontal Cortex

**DOI:** 10.29252/NIRP.BCN.9.2.135

**Published:** 2018

**Authors:** Hamidreza Ghaffari, Ali Yoonessi, Mohammad Javad Darvishi, Akbar Ahmadi

**Affiliations:** 1. Department of Neuroscience and Addiction Studies, School of Advanced Technologies in Medicine, Tehran University of Medical Sciences, Tehran, Iran.; 2. Department of Biomedical Engineering, Faculty of Engineering, Shahed University, Tehran, Iran.

**Keywords:** Transcranial Direct Current Stimulation (tDCS), Dorsolateral Prefrontal Cortex (DLPFC), Obsessive-Compulsive Disorder (OCD), Electroencephalogram (EEG)

## Abstract

**Introduction:**

Transcranial Direct Current Stimulation (tDCS) has been used as a non-invasive method to increase the plasticity of brain. Growing evidence has shown several brain disorders such as depression, anxiety disorders, and chronic pain syndrome are improved following tDCS. In patients with Obsessive-Compulsive Disorder (OCD), increased brain rhythm activity particularly in the frontal lobe has been reported in several studies using Eectroencephalogram (EEG). To our knowledge, no research has been done on the effects of electrical stimulation on brain signals of patients with OCD. We measured the electrical activity of the brain using EEG in patients with OCD before and after tDCS and compared it to normal participants.

**Methods:**

Eight patients with OCD (3 males) and 8 matched healthy controls were recruited. A 64-channel EEG was used to record a 5-min resting state before and after application of tDCS in both groups. The intervention of tDCS was applied for 15 minutes with 2 mA amplitude where anode was placed on the left Dorsolateral Prefrontal Cortex (DLPFC) and cathode on the right DLPFC.

**Results:**

In line with previous studies, the results showed that the power of Delta frequency band in OCD patients are significantly higher than the normal group. Following anodal tDCS, hyperactivity in Delta and Theta bands declined in most channels, particularly in DLPFC (F3, F4) and became similar to normal signals pattern. The reduction in Delta band was significantly more than the other bands.

**Conclusion:**

Anodal tDCS over the left DLPFC significantly decreased the power of frequency bands of Delta and Theta in Patients with OCD. The pattern of EEG activity after tDCS became particularly similar to normal, so tDCS may have potential clinical application in these patients.

## 1. Introduction

Obsessive-Compulsive Disorder (OCD) is a neuropsychiatric disorder with a lifelong predominance of 2% to 4% which happens at a 1:1 male to female ratio (Olbrich et al., 2013). It is characterized by regressive sudden thoughts (obsessions) and repetitive behaviors. It has been postulated that OCD is largely caused by the dysfunction of Cortico-Striato-Thalamo-Cortical (CSTC) circuitry, including the Orbitofrontal Cortex (OFC), medial prefrontal cortex, basal ganglia, and the Dorsolateral Prefrontal Cortex (DLPFC) (Tortella, 2015). Supporting that, imaging studies in OCD reveal abnormalities in relevant structures like OFC, limbic system, and DLPFC (Velikova et al., 2010). Also based on Liu et al. (2017) study, cognitive dysfunction and compulsive behaviors in patients with OCD are associated with abnormalities within the DLPFC. Their results showed that the resistant OCD subgroup were presented with distractibility and inadequate inhibitory activity. The researchers attributed it to abnormal activity within the fusiform gyrus and DLPFC. Taken together, their findings suggest that dysregulation of these areas plays key roles in the pathophysiology of OCD.

Russell et al. (2003) study on patients with OCD demonstrate that neurobiological abnormalities in the pre-frontal cortex are involved in the pathogenesis of the OCD. These outcomes provide new evidence of localized functional neurochemical marker changes in the left DLPFC in OCD. High levels of N-Acetyl-Aspartate (NAA) in left DLPFC can point to glial hypoplasia, neuronal hypertrophy, and or unusual pruning of neural brain elements in DLPFC (Russell et al., 2003).

Many investigations on the power of Electroencephalogram (EEG) in OCD concluded with conflicting results. Some of them reported an increase in alpha and beta action in the central channels (Prichep et al., 1993), while others reported a decline of both delta and beta (Kuskowski et al., 1993) or increased delta and diminished alpha wave (Ischebeck, Endrass, Simon, & Kathmann, 2014). Another investigation by Desarkar, Sinha, Jagadheesan, & Nizamie, (2007) demonstrates that patients with OCD have fundamentally higher power in contrast with the control group, mostly in theta band and were predominantly left sided in fronto-temporo-parietal districts in delta and alpha bands, and just left sided in the frontal area in beta 2 (12-18 Hz) band. Nevertheless, most studies in this context agree upon that OCD patients at least in one or both band(s) (theta & delta bands) have higher power in contrast with the control group (Karadag et al., 2003; Desarkar et al., 2007; Ischebeck et al., 2014).

Recent functional imaging techniques (low-resolution electromagnetic tomography and variable resolution electromagnetic tomography) based on quantitative EEG have shown expanded beta action in the cingulate gyrus in patients with OCD compared with the control group (Velikova et al., 2010). In addition, increased alpha activity in the corpus striatum, thalamus, temporoparietal and orbitofrontal areas has been reported in OCD responders to selective serotonin reuptake inhibitors (SSRI) whereas a lower frontal beta activity was related to a better treatment response (Fontenelle, Mendlowicz, Ribeiro, Piedade, & Versiani, 2005). On the other hand, non-invasive brain stimulation tools, such as Transcranial Magnetic Stimulation (TMS), Transcranial Direct Current Stimulation (tDCS), and Transcranial Alternating Current Stimulation (tACS) have acquired growing interest as efficient tools to modify cortical excitability and activity.

Some studies demonstrated that tDCS can be used effectively in modulating cortical excitability, in turn affecting a wide range of cognitive functions and sensorimotor in pathological and healthy human brains (Romero Lauro et al., 2014).

Investigation on animals showed that DC stimulation may increase, decline, or even silence firing of neurons in the primary motor region (M1). Another study shows that cathodal polarization of the motor cortex diminishes the size of the TMS and induces Motor Evoked Potentials (MEP) in humans. Conversely, anodal stimulation expands the size of the MEP (to 150%). Thus different polarizations have a distinguished impact on cortical excitability. The continuity of these electrophysiological effects outlasted the duration of the stimulation, in a 9–13 minutes session of 1 mA polarization, the effects continued for up to 90 min after the session (Zhang et al., 2011). It is suggested that tDCS is efficient in the treatment of patients with neurological and neuropsychiatric disorders like depression (Brunoni, Ferrucci, Fregni, Boggio, & Priori 2012), Parkinson (Benninger et al., 2010), schizophrenia (Vercammen et al., 2011; Brunelin et al., 2012), and addiction. Since OCD is identified with hyperactivity of the orbitofrontal subcortical circuit, tDCS can possibly help these patients by adjusting excitability of the dorsolateral or orbitofrontal prefrontal cortex.

It is well-founded to suppose that tDCS might induce a change in the DLPFC activity, a crucial area in the cortico-subcortical, anxiety-related neural network. Neuroimaging studies have demonstrated that anxious individuals exhibit lower activation in the left DLPFC during inhibition tasks. One study on patients with OCD showed that when high-frequency rTMS was applied on the left DLPFC for up to four weeks, a significant decline in whole Yale-Brown Obsessive Compulsive Disorder Scale (Y-BOCS) scores was seen, however not after controlling for depression. Furthermore, the studies demonstrate that anodal stimulation over the left DLPFC ameliorate performance in solving insight problems that are associated with OCD symptoms. Also, recent studies show that tACS might have therapeutic effects on psychiatric diseases. A case report by Klimke, Nitsche, Maurer, & Voss (2016) shows that few tACS sessions at fronto-temporal area on 7 patients with OCD who were treatment resistant, could considerably improve their symptoms.

Many investigations have explored the effect of tDCS in OCD. A case report indicates that after ten daily sessions of 2 mA cathodal stimulation for 20 min on the DLPFC, no progressions were seen in OCD symptoms with sham or real stimulation. However, it decreased anxiety and depressive symptoms (Bais, Figee & Denys, 2014).

Several studies have recommended that tDCS could be beneficial in reducing symptoms in patients with treatment-resistant OCD (Volpato et al., 2013; Narayanaswamy et al., 2015; Mondino, Haesebaert, Poulet, Saoud, & Brunelin 2015). Although suitable target locations and stimulation parameters have remained under debate. Similarly, a case-report by D’Urso et al. (2015) on a patient with OCD shows that 10 sessions cathodal stimulation (2 mA/20 min tDCS) of the pre-supplementary motor area (pre-SMA) results in a dramatic clinical improvement and generally 30% decrease in baseline symptoms severity score on the Y-BOCS.

A recent study conducted by Bation, Poulet, Haesebaert, Saoud, & Brunelin (2016) shows that 10 sessions of 2 mA cathodal stimulation over the left OFC on 8 patients with treatment-resistant OCD, has reduced OCD symptoms in Y-BOCS score. Furthermore, tDCS had no effect on depressive symptoms in this investigation.

This study aimed to investigate the impact of tDCS, in the power spectra in patients with OCD and compare it with normal subjects by a high-resolution EEG method. It has been hypothesized that anodal electrical stimulation of the DLPFC will lessen power frequency bands, particularly in delta and theta bands in patients with OCD.

## 2. Methods

### 2.1. Study participants

Based on DSM-IV (American Psychiatric Association, 1994) criteria, eight patients were diagnosed with OCD by qualified clinical psychologists and eight healthy controls matched for sex, age, and handedness were recruited, too. Exclusion criteria consisted of having history of any seizures and other neurological disorders, mood disorders, chronic medical disease, severe head injury and any neurosurgical experiences. Moreover, the control group should not have any history of mental problem. For this purpose, we used General Health Questionnaire (GHQ-28) (Shamasunder, Sriram, Raj, & Shanmugham, 1986) to screen the control group. The statistical sample of the research in the control group included male and female students of the Institute of Cognitive Science, aged 21 to 34 years (with a mean age of 27.5), and the intervention group of male and female patients (OCD group) from the consulting center of the National Iranian Oil Company, aged 22 to 36 years (with a mean age of 29). In this study, a total of 16 people attended, nine men and seven women.

During EEG recording, six patients were drug-free while two of them were under SSRIs medication. Clinical record in the patient group contained OCD’s age of onset, duration, and symptom seriousness. Symptom seriousness was evaluated by the Yale–Brown Obsessive Compulsive Scale (Y-BOCS) (Goodman et al., 1989). All patients after full explanation were consented verbally and written to participate in the experiment. This research was performed at the Cognitive Laboratory of Institute for Cognitive Science Studies (ICSS), Tehran, Iran.

### 2.2. Study methods

In this study, 64-channel amplifier device (ANT Neuro Netherlands, 64 ES-410) were used to record brain signals. The brain signals of the participants were recorded for 5 minutes before electrical stimulation (the baseline). Then electrical stimulation (2 mA current intensity) was applied for 15 minutes. The anodal electrode was placed over the DLPFC on the left hemisphere. Cathode electrode was placed in the right area of DLPFC. At the end, we recorded brain signals of the subjects for 5 minutes to observe the effects of electrical stimulation and compare it with the baseline.

### 2.3. EEG data acquisition

Ten-twenty International System for electrode placement has been used in which the impedance was kept down below 5 kΩ. The rate of sampling was 512 Hz. High and low frequency filters, with a notch filter of 50 Hz, were adjusted at 0.5 Hz and 30 Hz, respectively. All people were requested not to smoke or take caffeine 3 hours before the recording. The whole experiment took about 25 minutes in a semi-dark room with the subject’s eyes closed.

### 2.4. EEG data processing

We used EEGLAB toolbox in Matlab for data analysis (Delorme & Makeig, 2004). Four channels had to be excluded from the original arrangement of 64 channels because it was impossible to get two minutes of artifact-free data out of them; these channels were F4, FC5, FC4, CP4. Ultimately, 60 electrodes were selected to analyze power spectrum (Table 1) and calculated by using the Fast Fourier Transform (FFT) function implemented in EEGLAB study.

**Table 1.** EEG protocol in this study

**Design**	**EEG Protocol**					

	**Number of Channels**	**EEG Recording**	**System for Electrode Placement**	**Impedance Maintenance**	**Sampling Rate**	**Low and High Frequency Filters**
	64	Eye-closed 10–20	International system	5 kΩ	512 Hz/per channel	0.5–30 Hz

### 2.5. Data analysis

Statistical analysis was done using the EEGLAB Matlab toolbox. Independent t test was used to compare the differences of spectral power values for delta and theta bands between patient and control group in the baseline and after tDCS and paired t test on patients for comparison between pre- and post-tDCS, across 60 channels. The level of significance for power data taken was P<0.05. In this study, we have carried out False Discovery Rate (FDR) procedure based on Holms correction. Under FDR, a standard reformed threshold is applied to all P values. This threshold is the last remarkable threshold computed using Holms correction. By using this method, statistical assessment is accomplished independently for each P value.

### 2.6. Transcranial direct current stimulation

The electrical stimulation device used in this research was ActivaDose Iontophoresis device (Active Tek Co). The size of the electrodes in this study was 5×5 cm square, which was in the solution of 9% chloride sodium. The anodal electrode was placed on the left DLPFC and the cathodal electrode on the right DLPFC (Table 2). Details of the stimulation protocols as well as effects of stimulation are presented.

**Table 2.** tDCS protocol

**Design**	**Stimulation Protocol**						

	**Polarity**	**Stimulation Electrode Position**	**Reference Electrode Position**	**Current Strength (mA)**	**Electrode Size (cm^2^)**	**Duration (min)**	**Current Density (mA/cm^2^)**
	A/C	Left/right DLPFC	F3/F4 DLPFC	2	25	15	0.06

A: Anodal; tDCS, C: Cathodal tDCS.

Stimulation target areas are described according to the international 10–20.

## 3. Results

### 3.1. Comparison of baseline EEG between OCD and normal subjects

**Figure 1.**
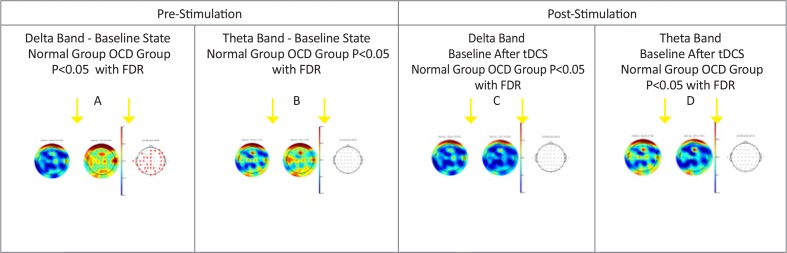
Brain maps showing differences between pre-stimulation in both group and post-stimulation in OCD patients Comparison of power spectrum of normal subjects with OCD patients before (A, B) and after stimulation (C, D). A: Delta band power; B: Theta band power (both pre-stimulation); C: Delta band power; D: Theta Band power (both post-stimulation). No significant difference was observed between C and D. Black dots show EEG channel locations and the power of the EEG channels color-coded and plotted at the corresponding location on the scalp. The third top plot related to P value that red dots specify the significant channels. Also, the color bar on the corner of the brain maps shows the signal strength spectrum in each position.

**Figure 2.**
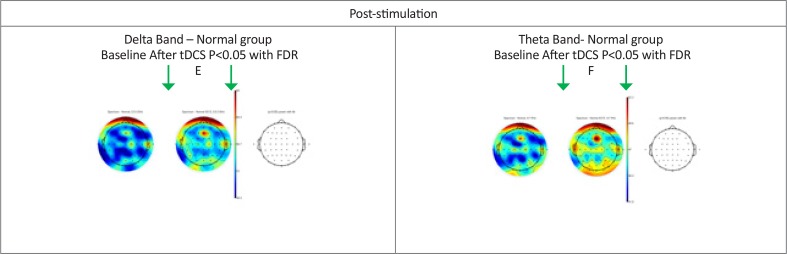
Brain maps showing differences between pre and post-stimulation in normal subjects E: Delta band power; F: Theta band power (both post-stimulation). Black dots show EEG channel locations and the power of the EEG channels color-coded and plotted at the corresponding location on the scalp. The third top plot related to P value that red dots specify the significant channels. Also, the color bar on the corner of the brain maps shows the signal strength spectrum in each position.

**Figure 3.**
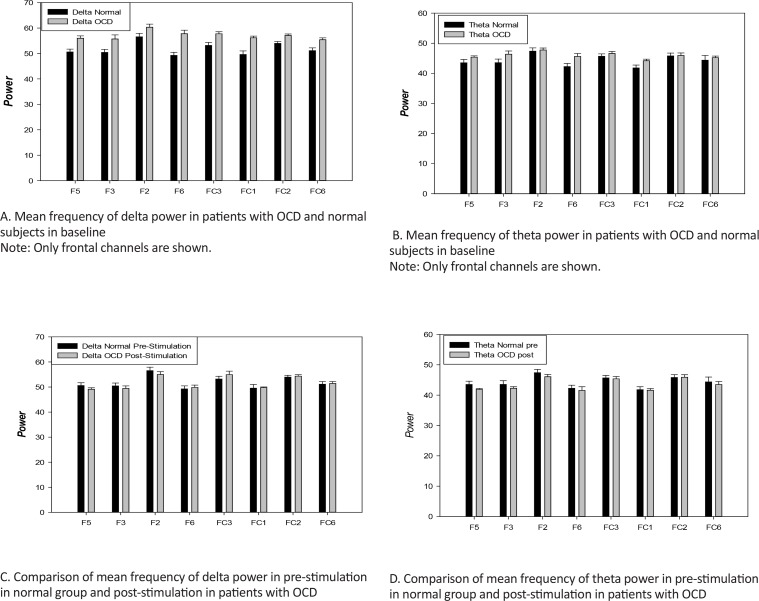
Mean frequency bands (Delta & Theta) in pre- and post-stimulation in OCD patients and control group Graph (A) shows that the power of delta (0.5–3.5 Hz) band in pre-stimulation in OCD patients was higher than the control group in each of the eight selected channels (P<0.05). Graph (B) shows that the power of theta (4–7.5 Hz) band, although most of the channels had higher power than the control group, no striking difference was observed in any of the selected channels. Graphs (C and D) prove that the power frequency bands of OCD patients became similar to the control group’s baseline with regard to the delta and theta bands. No remarkable change was observed in the delta and theta bands.

The power of delta (0.5–3.5 Hz) frequency band in patients with OCD was much higher than the control group in most channels (P<0.05). This difference is more widespread in the delta band and particularly in the left side (Figure 1, A and B). Figure 2 shows the comparison of the power of frontal channels in the delta band between the control and OCD group. For the theta band, although most of the channels had higher power than the normal group, no significant difference was observed in any of the channels. Figure 3 shows comparison of power of frontal channels in the theta band between the control and OCD group. In the alpha band (8–12 Hz), a similar pattern was visible. With regard to beta 1(12.5–18Hz), beta 2(18.5–24), and beta 3(24.5–30) no significant difference was observed (except for one channel in beta 2 and 3). Furthermore, the results of statistical comparisons in delta and theta power (μV2/Hz) in these groups (Only frontal channels) has shown in Table 3 and 4.

**Table 3.** Group comparison in delta power (μV2/Hz) in patients with OCD and controls in baseline state (Only frontal channels are shown)

**Channel**	**OCD (n=8)**		**Normal (n=8)**		**t**	**P**

	**Mean**	**SD**	**Mean**	**SD**		
F5	55.98	2.67	50.58	3.19	3.43	0.005
F3	55.70	4.52	50.42	3.34	2.40	0.039
F2	60.31	3.42	56.54	3.90	1.90	0.082
F6	57.75	3.98	49.25	3.44	4.17	0.001
FC3	57.71	2.30	53.18	3.19	3.07	0.009
FC1	56.16	1.92	49.56	4.08	4.02	0.002
FC2	57.07	2.70	53.97	2.01	2.36	0.042
FC6	55.39	1.23	51.11	3.66	3.07	0.013

SD: Standard Deviation; OCD: Obsessive Compulsive Disorder

* P<0.05.

**Table 4.** Group comparison in theta power (μV2 /Hz) in patients with OCD and controls in baseline state

**Channel**	**OCD (n=8)**		**Normal (n=8)**		**t**	**P**

	**Mean**	**SD**	**Mean**	**SD**		
F5	45.38	1.25	43.50	3.13	1.54	1.54
F3	46.37	3.01	43.52	3.49	1.63	0.12
F2	47.76	1.87	47.37	3.09	0.29	0.77
F6	45.63	2.63	42.25	2.92	2.26	0.04
FC3	46.60	1.89	45.66	2.30	0.83	0.42
FC1	44.18	1.54	41.78	2.79	2.04	0.06
FC2	45.91	2.44	45.80	2.67	0.08	0.93
FC6	45.21	1.53	44.35	4.55	0.49	0.63

### 3.2. Comparing before and after tDCS in patients with OCD and control subjects

The power spectrum in patients with OCD reduced significantly compared to the baseline status after tDCS. The power spectrum showed a significant reduction after tDCS in the delta and theta bands in most channels. Furthermore, the power of beta bands also decreased. Whereas in normal subjects no significant difference was observed in the baseline status after tDCS (Figure 2, E and F). In addition, the results of statistical comparisons in delta and theta power (μV2/Hz) before and after tDCS in both groups (Only frontal channels) has shown in Table 5 and 6.

**Table 5.** Group comparison in delta power (μV2 /Hz) in pre-stimulation in the control group & post-stimulation in OCD group

**Channel**	**OCD (n=8)**		**Normal (n=8)**		**t**	**P**

	**Mean**	**SD**	**Mean**	**SD**		
F5	49.10	1.72	50.58	3.19	1.11	0.28
F3	49.45	2.83	50.42	3.34	0.58	0.56
F2	54.98	3.25	56.54	3.90	0.81	0.43
F6	49.86	2.63	49.25	3.44	0.37	0.71
FC3	54.94	2.78	53.18	3.19	1.09	0.29
FC1	49.76	0.75	49.56	4.08	0.13	0.89
FC2	54.34	1.68	53.97	2.01	0.37	0.71
FC6	51.45	2.10	51.11	3.66	0.21	0.83

**Table 6.** Group comparison in theta power (μV2 /Hz) in pre-stimulation in the control group and post-stimulation in OCD patients

**Channel**	**OCD (n=8)**		**Normal (n=8)**		**t**	**P**

	**Mean**	**SD**	**Mean**	**SD**		
F5	41.91	0.87	43.50	3.13	1.36	2.07
F3	42.26	1.49	43.52	3.49	0.91	0.38
F2	46.05	2.18	47.37	3.09	0.92	0.37
F6	41.57	3.39	42.25	2.92	0.39	0.70
FC3	45.37	2.16	45.66	2.30	0.24	0.81
FC1	41.61	1.58	41.78	2.79	0.14	0.88
FC2	45.89	2.37	45.80	2.67	0.06	0.94
FC6	43.49	2.79	44.35	4.55	0.43	0.66

### 3.3. Comparing patients with OCD after tDCS with normal subjects’ baseline

Interestingly, the power frequency bands of patients with OCD became similar to the normal group’s baseline with respect to the delta and theta bands. No significant difference was observed in the delta band (Figure 1, C). Theta band power graph is also shown (Figure 1, D). Comparison of power of frontal channels in the delta and theta bands between normal (pre-stimulation) and OCD (post-stimulation) group are shown in Figure 3c and Figure 3d.

## 4. Discussion

Our results show that frequency power of delta and theta bands in patients with OCD is higher than the control group. Our baseline EEG finding is consistent with results of Desarkar et al. (2007) study. Moreover, the results of our study show that the power spectrum in OCD patients reduced appreciably compared to the baseline status after tDCS. Interestingly, after stimulation, the power frequency bands of patients with OCD became similar to the normal group’s baseline with respect to the delta and theta bands. To our knowledge, no research has been done to investigate the effects of electrical stimulation on brain signals of OCD patients. Some investigations demonstrated that tDCS can modify synaptic neuromodulator concentration. Furthermore, altered synaptic plasticity has been associated with neuropsychiatric disorders and cognitive impairment (Podda et al., 2016). Thus targeting synaptic plasticity may provide a major breakthrough for therapeutic interventions.

Several studies provided molecular and functional evidence linking synaptic plasticity in some brain regions to specific motor and cognitive functions (Podda et al., 2016). Additionally, based on the evidence PFC neurons have the cellular machinery of synaptic plasticity and show stable changes in neural activity associated with different cognitive processes. Furthermore, deficiencies in the mechanisms of synaptic plasticity induction in PFC could be involved in the pathophysiology of neurological and psychiatric disorders, such as anxiety disorders, mood disorders, schizophrenia, and Alzheimer disease (Crabtree & Gogos, 2014).

Empirically, changes in neural oscillations have been found in all major psychiatric diseases (Buzsáki & Watson, 2012). Therefore, tDCS could produce clinical palliation by strengthening or weakening oscillatory activities within brain areas (Das, Holland, Frens, & Donchin, 2016). Several studies recommend that tDCS could be beneficial in reducing symptoms in patients with treatment-resistant OCD patients (Bation et al., 2016; Mondino et al., 2015; Narayanaswamy et al., 2015; Volpato et al., 2013).

Some investigations have focused on mechanisms of neural plasticity on the specific brain waves’ role in modulating neural plasticity (Diekelmann & Born, 2010). Current studies concentrate on comprehension the role of synaptic and neuronal plasticity in generating specific brain rhythms related with particular physiologic state and function (Liu, Bartsch, Lin, Mantegna, & Ivanov, 2015). Studies show the role of delta wave in neural plasticity processes which are taking place during the awakening mode (Assenza, Pellegrino, Tombini, Di Pino, & Di Lazzaro 2015). In addition, NMDA receptor activation, which can be considered as a part of glutamate receptor, causes the stimulation of Long-Term Potentiation (LTP), which can occur after electrical stimulation. This excitability of cortical plasticity can cause changes in delta waves which mainly occur in stimulation areas, but are not necessarily limited to the location of the stimulation (Assenza et al., 2015). Therefore, delta wave seems to play a significant role in the plasticity of the brain.

There is substantial evidence about the relationship between frequency fluctuations of delta, theta, and alpha bands in the brain with cognitive processing (Basar, Başar Eroglu, Karakaş, & Schürmann, 2001). Theta band reflects integrated cognitive processing; delta frequency band is also involved in a similar process (Basar et al., 2001). Increasing frequency band power in obsessed patients associates with cognitive processing, particularly that this increase is mostly seen in the frontal area. It has been suggested that DLPFC integrates information about motivation, attention, and problem solving and uses it in the response process (Cooke, 2006). Also mounting evidence shows that stimulating the DLPFC area improves cognitive functions in healthy subjects.

Moreover, research evidence suggests that changes in brain signals can be effective in improving symptoms in clinical disorders. EEG biofeedback investigations on OCD patients show that changes in the brain electrical activities in patients can reduce the symptoms and eventually lead to successful treatment. Hammond (2005) study on OCD patients shows that neurofeedback has been able to improve the scores of patients on the Y-BOCS and the Padua Inventory normalized following treatment. Furthermore, another neurofeedback investigation by Dong and Bao (2005) on students diagnosed with great levels of anxiety indicated that when biofeedback sessions were implemented on them, they showed remarkable betterment in depressive signs, somatization, and anxiety-related symptoms compared with the control group. Also many similar studies on clinical disorders like posttraumatic stress disorder, generalized anxiety disorder, attention deficit hyperactivity disorder, depression have been carried out with similar results (Hammond, 2005). Therefore, it is very likely that tDCS can improve symptoms of patients with OCD.

Based on our intervention, anodal tDCS over the left DLPFC area significantly decreases the power in delta and theta bands in patients with OCD. This intervention made the power spectrum similar to the normal subjects. This important effect holds promises for a novel treatment for these patients. Further research can explore whether these changes in the EEG are related with clinical improvement. However, our sample size was small and this study can be improved using more subjects. In addition, we did not evaluate the clinical consequence of the electrical stimulation and therefore, further research is needed to evaluate the clinical efficacy of the electrical stimulation in patients with OCD.
